# Bacillus Calmette–Guérin-Induced Trained Immunity Is Not Protective for Experimental Influenza A/Anhui/1/2013 (H7N9) Infection in Mice

**DOI:** 10.3389/fimmu.2018.00869

**Published:** 2018-04-30

**Authors:** L. Charlotte J. de Bree, Renoud J. Marijnissen, Junda M. Kel, Sietske K. Rosendahl Huber, Peter Aaby, Christine Stabell Benn, Marcel V. W. Wijnands, Dimitri A. Diavatopoulos, Reinout van Crevel, Leo A. B. Joosten, Mihai G. Netea, John Dulos

**Affiliations:** ^1^Department of Internal Medicine, Radboud University Medical Center, Nijmegen, Netherlands; ^2^Radboud Centre for Infectious Diseases (RCI), Radboud University Medical Center, Nijmegen, Netherlands; ^3^Research Center for Vitamins and Vaccines, Bandim Health Project, Statens Serum Institut, Copenhagen, Denmark; ^4^Odense Patient Data Explorative Network, University of Southern Denmark, Odense University Hospital, Odense, Denmark; ^5^Department of Immunology, Triskelion B.V., Zeist, Netherlands; ^6^Laboratory of Pediatric Infectious Diseases, Radboud Institute for Molecular Life Sciences, Radboud University Medical Center, Nijmegen, Netherlands; ^7^Department for Genomics and Immunoregulation, Life and Medical Sciences Institute (LIMES), University of Bonn, Bonn, Germany; ^8^Aduro Biotech Europe, Oss, Netherlands

**Keywords:** avian influenza A/Anhui/1/2013 (H7N9), bacillus Calmette–Guérin, trained immunity, innate immune memory, oseltamivir

## Abstract

Avian influenza A of the subtype H7N9 has been responsible for almost 1,600 confirmed human infections and more than 600 deaths since its first outbreak in 2013. Although sustained human-to-human transmission has not been reported yet, further adaptations to humans in the viral genome could potentially lead to an influenza pandemic, which may have severe consequences due to the absence of pre-existent immunity to this strain at population level. Currently there is no influenza A (H7N9) vaccine available. Therefore, in case of a pandemic outbreak, alternative preventive approaches are needed, ideally even independent of the type of influenza virus outbreak. Bacillus Calmette–Guérin (BCG) is known to induce strong heterologous immunological effects, and it has been shown that BCG protects against non-related infection challenges in several mouse models. BCG immunization of mice as well as human induces trained innate immune responses, resulting in increased cytokine responses upon subsequent *ex vivo* peripheral blood mononuclear cell restimulation. We investigated whether BCG (Statens Serum Institut-Denmark)-induced trained immunity may protect against a lethal avian influenza A/Anhui/1/2013 (H7N9) challenge. Here, we show that isolated splenocytes as well as peritoneal macrophages of BCG-immunized BALB/c mice displayed a trained immunity phenotype resulting in increased innate cytokine responses upon *ex vivo* restimulation. However, after H7N9 infection, no significant differences were found between the BCG immunized and the vehicle control group at the level of survival, weight loss, pulmonary influenza A nucleoprotein staining, or histopathology. In conclusion, BCG-induced trained immunity did not result in protection in an oseltamivir-sensitive influenza A/Anhui/1/2013 (H7N9) challenge mouse model.

## Introduction

Since its first outbreak in China in 2013 until December 2017, avian influenza A (H7N9) has been responsible for 1,565 confirmed human cases, including 612 deaths (1). Infections are characterized by a high incidence of pneumonia, respiratory failure, and acute respiratory distress syndrome. Avian influenza A (H7N9) is transmitted after contact with live poultry or exposure to contaminated environments. Apart from some small reported clusters, sustained human-to-human transmission is rare (2). Nevertheless, because immunity to this strain at population level is negligible, public authorities fear that possible additional mutations and reassortment with circulating other human-adapted influenza viruses may enable human–human transmission and infection, which could potentially lead to a severe H7N9 influenza pandemic (3).

Vaccine development is part of pandemic preparedness strategies. Although numerous inactivated and live attenuated H7 vaccines are being developed, the immunogenicity of non-adjuvanted or aluminum hydroxide-adjuvanted candidate H7 vaccines is low (4, 5). Therefore, novel approaches for protection against influenza A (H7N9) are needed. Adjuvanted (MF59 or AS03) vaccines have shown to elicit enhanced immunogenicity against H7N9 (6–8). An alternative approach is to make use of non-specific beneficial effects of already existing vaccines, *via* the induction of the newly described process of trained immunity. Bacillus Calmette–Guérin (BCG) immunization confers broad heterologous protection after vaccination. Thereby, BCG could potentially offer directly available protection in case of an outbreak, independent of the type of influenza virus outbreak.

Bacillus Calmette–Guérin, the widely used live attenuated vaccine against tuberculosis, has long been known for its immune modulatory effects. Upon its introduction in Sweden in 1932, the Swedish physician Carl Näslund observed a strong decrease in childhood mortality in the first year of life in the provinces in which BCG was introduced (9). This improvement could not be explained by prevention of tuberculosis alone. Similar observations were made several times upon introduction of BCG vaccination in other countries and were validated in randomized controlled trials (10, 11). Non-specific beneficial effects after BCG immunization have been demonstrated in several mouse studies, such as *Plasmodium* (12–14), *Schistosoma* (15), and disseminated *Candida* infection models (16). Moreover, it has been shown that BCG administration improves the outcome of a lethal challenge with the seasonal influenza A/Puerto Rico/8/34 (H1N1) in an experimental mouse model (17). The heterologous protective effects of BCG vaccination are at least partially explained by the induction of trained immunity: monocytes of BCG-vaccinated individuals display increased immune responsiveness, such as enhanced cytokine production upon restimulation with unrelated pathogens and toll-like receptor (TLR) ligands, a process which is dependent on epigenetic and metabolic rewiring of myeloid cells (16, 18). In epidemiological studies, the non-specific effects of BCG vaccination are most pronounced in the first year of life, suggesting that trained immunity is most strongly activated during this first year (10, 19). This is in line with the study by Kleinnijenhuis et al. showing 1-year duration for trained immunity (20). Moreover, BCG vaccination resulted in heterologous T-helper cell 1 (Th1) and T-helper cell 17 (Th17) immune responses and enhanced immunogenicity after subsequent influenza vaccination in healthy volunteers (20, 21). Recently, we have shown that BCG vaccination resulted in reduced peak viremia after subsequent yellow fever vaccination of healthy volunteers, a process depending on the induction on monocyte responses, rather than T-cell heterologous immunity (22).

We therefore hypothesized that BCG vaccination may induce non-specific protection against influenza A (H7N9) infection, a strategy that may offer important public health benefits. In this study, we assessed the effects of BCG immunization in an experimental lethal avian influenza A/Anhui/1/2013 (H7N9) infection in BALB/c mice.

## Materials and Methods

### H7N9 Influenza Virus Stock Preparation and TCID_50_ Determination

A/Anhui/1/2013 (H7N9) seed virus was obtained from the National Institute for Biological Standards and Control (UK). A new influenza A/Anhui/1/2013 (H7N9) virus stock was obtained after propagation in 11-day-old embryonic chicken eggs for 32 h at 37°C. Aliquots were stored at <−70°C and were confirmed to be negative for endotoxin and mycoplasma. No novel mutations were introduced in the hemagglutinin and neuraminidase segments. The homology compared to the reference amino acid sequence (GenBank) was >99%. For the 50% tissue culture infectious dose (TCID_50_) assay, Madin–Darby Canine Kidney (MDCK) cells (ATCC CCL-34) were cultured in Dulbecco’s Modified Eagle Medium (DMEM) (Gibco, Life technologies) with Glutamax (Gibco, Life technologies), 10% fetal calf serum (FCS) (Lonza, Switzerland), supplemented with 100 U/ml penicillin, 100 µg/ml streptomycin (Gibco, Life technologies), and 1× non-essential amino acids (Gibco, Life technologies) at 5% CO_2_ and 37°C. One day prior to the start of the assay, 30,000 cells per well were seeded in 96-well flat-bottom plates (Corning) and incubated overnight (37°C, 5% CO_2_). The cells were washed and incubated with serial dilutions of the influenza virus in culture medium (DMEM + glutamax, supplemented with 100 U/ml penicillin, 100 µg/ml streptomycin, 0.0004% trypsin-EDTA). After 7 days of incubation at 34°C, wells were scored for the cytopathic effect (CPE). The TCID_50_ titer was calculated using the Reed–Muench method (23).

### Animal Ethics Statement

Animals experiments were performed in accordance with the guidelines of the European Communities (Directive 2010/63/EU) and Dutch legislation (The experiments on Animals Act, 1997). The animal experimental protocols were approved by an independent Animal Ethics Committee (TNO, Zeist, the Netherlands) under project license 3387 and performed in the AAALAC accredited animal facility of Triskelion. All animals were housed in a temperature and light-cycle controlled facility with unlimited access to food and water. All procedures involving live H7N9 viruses, including the animal experiments, were carried out in a biosafety level 3 (BSL-3) containment facility at Triskelion. The animals were monitored for clinical signs of influenza disease twice daily with intervals of at least 5 h. All observations, including behavioral aspects were recorded. If lethargy was observed longer than 48 h, the animal was euthanized (humane endpoint).

### Influenza Challenge Model

Female BALB/cAnNCrl (BALB/c) mice were obtained (Charles River, Germany) and maintained under SPF conditions. At commencement of the experiments, animals were 6–8 weeks old. All experiments were performed with 8–10 mice per group. On day 0, mice were challenged intranasally (i.n.) with influenza A/Anhui/1/2013 (H7N9) diluted in 50 µl phosphate buffered saline (PBS) under anesthesia with ketamine/xylazine (50 and 5 mg/kg, respectively). To determine the 50% mouse lethal dose (MLD_50_), six groups of eight animals were challenged with 7.31, 6.17, 5.02, 3.87, 2.73, or 1.58 log_10_ TCID_50_ per mouse and monitored until they succumbed to infection or until scheduled sacrifice 14 days post-infection. The MLD_50_ was calculated using the Spearman–Kärber method. Throughout the experiment, clinical signs were monitored twice daily and body weight was recorded once daily until death or scheduled sacrifice.

As a reference control for the lethal challenge model, two additional groups received the neuraminidase inhibitor oseltamivir phosphate (Tamiflu^®^; Roche, Switzerland) dissolved in sterile water (Fresenius Kabi, the Netherlands) and stored at 2–10°C until use. One group of animals was treated with 100 mg/kg twice daily *per os* (p.o.) starting 1 h prior to the challenge on day 0 continuing until day 4, while another group received oseltamivir on days 1–5. The control group was treated with vehicle PBS (Gibco, Life technologies) p.o. on days 0–4. For evaluation of the effect of BCG, animals received either 750 µg BCG (Danish strain 1331; Statens Serum Institut, Denmark) dissolved in 200 µl PBS intravenously (i.v.), containing 2–8 × 10^6^ colony-forming units, or PBS i.v. on day –7. To demonstrate induction of trained innate immune responses after BCG vaccination, five animals per group were sacrificed prior to the influenza challenge on day 0, after which splenocytes and peritoneal macrophages were isolated for *ex vivo* restimulation experiments. On day 0, the animals were challenged with a 4MLD_50_ dose influenza A/Anhui/1/2013 (H7N9) and monitored until they succumbed to infection or until scheduled sacrifice at 21 days post-infection. Three days after viral challenge, eight animals per group were sacrificed, and lungs were collected and prepared for histopathological analysis.

### *Ex Vivo* Stimulations of Splenocytes and Peritoneal Macrophages

Spleen cells were isolated by gently squeezing spleens in a sterile 200 µM filter chamber. After washing with sterile PBS (1,200 rpm, 5 min, 4°C), cells were resuspended in 4 ml Roswell Park Memorial Institute (RPMI) 1640 culture medium (RPMI medium; Invitrogen, CA, USA) supplemented with 10% FCS. Cells were counted and concentrations were adjusted to 1 × 10^7^ cells/ml. Cells were cultured in 24-well plates (Greiner, the Netherlands) at 5 × 10^6^ cells/well, in a final volume of 1,000 µl and stimulated in duplo with RPMI, *Escherichia coli* lipopolysaccharide (LPS) (10 ng/ml, Sigma-Aldrich), phytohemagglutin (PHA) (10 µg/ml from *Phaseolus vulgaris*, Sigma-Aldrich). Poly I:C (50 µg/ml, Invivogen), heat-killed *Candida albicans* (1 × 10^6^ microorganisms/ml, strain UC820), heat-killed *Salmonella typhi* (1 × 10^7^ microorganisms/ml), or heat-killed *Staphylococcus aureus* (1 × 10^7^ microorganisms/ml). After 2 days of incubation, 500 µl supernatant was collected and the plates were incubated for another 3 days before the remaining supernatants were har-vested. The supernatants were stored at –80°C until levels of tumor necrosis factor-alpha (TNF-α), interferon (IFN)-α, IFN-γ, interleukin (IL)-17, and IL-22 were determined.

Peritoneal macrophages were isolated by injecting 5 ml of ice-cold sterile PBS in the peritoneal cavity. After centrifugation and washing, cells were resuspended in RPMI supplemented with 10 µg/ml gentamicin, 10 mM Glutamax, and 10 mM pyruvate. Cells were counted using a Z1 Coulter Particle Counter (Beckman Coulter, the Netherlands) and adjusted to 1 × 10^6^ cells/ml. Cells were cultured in 96-well round-bottom microtiter plates (Costar, Corning, the Netherlands) at 1 × 10^5^ cells/well, in a final volume of 200 µl. After 24 h of incubation with abovementioned stimuli in duplo {plus Pam3Cys [10 µg/ml, EMC microcollections (L2000)] instead of PHA} at 37°C in air and 5% CO_2_, the plates were centrifuged at 1,400 × *g* for 8 min, and the supernatants were collected and stored at –20°C until levels of TNF-α, IL-1α, IL-1β, IL-6, and IL-10 were determined.

### Quantification of Cytokine Concentrations

Cytokine concentrations were determined in supernatants using commercial enzyme-linked immunosorbent assay (ELISA) kits according to instructions of the manufacturer. TNF-α (R&D systems, MN, USA), IL-1α, IL-1β, IL-6, IL-10, IFN-α, and IFN-γ (Sanquin, the Netherlands) were determined in supernatants harvested after 2 days of culture. IL-17 and IL-22 (R&D systems) were determined in supernatants after 5 days of incubation.

### Histopathology and Immunohistochemistry

For histopathological examination, the lungs of both BCG and PBS treated animal groups were isolated 3 days post-challenge. Formalin fixed lung tissues were embedded in paraffin wax, sectioned at 4 µm, and stained with hematoxylin and eosin. Per animal, three consecutive HE-stained lung sections were semi-quantitatively scored for the presence of signs of inflammation, epithelial damage, and repair. Influenza nucleoprotein (NP) was visualized by immunohistochemistry using anti-influenza A NP antibody (Millipore, clone H16-L10-4R5, mouse IgG2a) as a measure for the amount of virus present in cells in the lung according to the protocol described in Rimmelzwaan et al. (24). NP-stained sections were semi-quantitatively scored for the presence of viral protein. All parameters were scored as absent (0), minimal (1), moderate (2), or marked (3).

### Statistical Analysis

Data were analyzed using Graphpad Prism 5.0 (La Jolla, CA, USA). *p-Value < 0.05, **p-value < 0.01. Cytokine data are shown as mean ± SEM. The survival proportion at day 21 after treatment was compared to the vehicle control group using a Fisher exact two-sided test, corrected for multiple comparisons. Survival times after viral challenge of the groups were compared using a log-rank test. Change in body weight was summarized as area under the curve (AUC) in which the last observed body weight was carried forward if a mouse died/was euthanized during the study. Briefly, the weight per mouse at day 0 was used as baseline and weight change was determined relative to baseline. The AUCs for each group were summarized as mean, SD, and adjusted *p*-value for comparison to the vehicle control group.

## Results

### Determination of Tissue Culture Infective Dose (TCID) and 50% MLD

**Figure 1:**
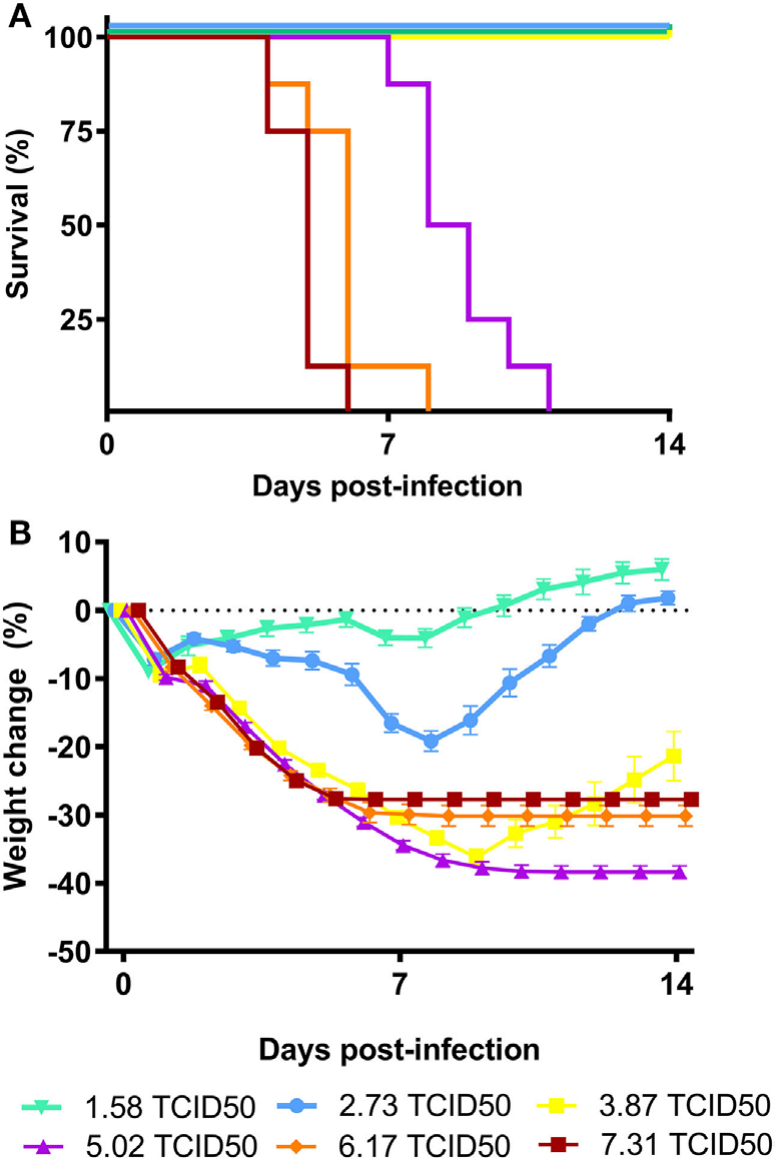
Virulence of influenza A/Anhui/1/2013 (H7N9) in BALB/c mice. Female BALB/c mice (*n* = 8 per group) were intranasally inoculated with serial dilutions of the A/Anhui/1/2013 (H7N9) influenza virus on day 0 and survival was monitored for 14 days. Kaplan–Meyer survival curve **(A)** and mean body weight change is depicted **(B)**. The MLD_50_ was calculated using the Reed–Muench method.

Based on the CPE observed in the MDCK cells incubated with the dose formulation of A/Anhui/1/2013 (H7N9), a TCID_50_ of 6.11 log_10_ TCID_50_/ml was calculated (data not shown). To assess the potency of BCG vaccination *in vivo*, we established a lethal A/Anhui/1/2013 (H7N9) influenza challenge model in female BALB/c mice, by inoculating groups of mice i.n. with a dose of 7.31, 6.17, 5.02, 3.87, 2.73, or 1.58 log_10_ TCID_50_/mouse. During the subsequent MLD experiment, progress of infection the body weights correlated with an increase in the number and severity of the clinical signs. Recovery of the animals was demonstrated by an increase in body weight and a decrease in clinical signs. Intranasal administration of influenza A/Anhui/1/2013 (H7N9) was lethal to female (BALB/c) mice, which has been shown previously (25). The MLD_50_ determined for the A/Anhui/1/2013 (H7N9) influenza was calculated at 4.45 log_10_ TCID_50_. The infectious dose for the subsequent experimental challenge infection study was set at 4MLD_50_, a dose where 0–10% of the animals were expected to survive the intranasal challenge with the virus (Figures 1A,B).

### Assessment of Oseltamivir Sensitivity of A/Anhui/1/2013 (H7N9) Challenge Model

**Figure 2:**
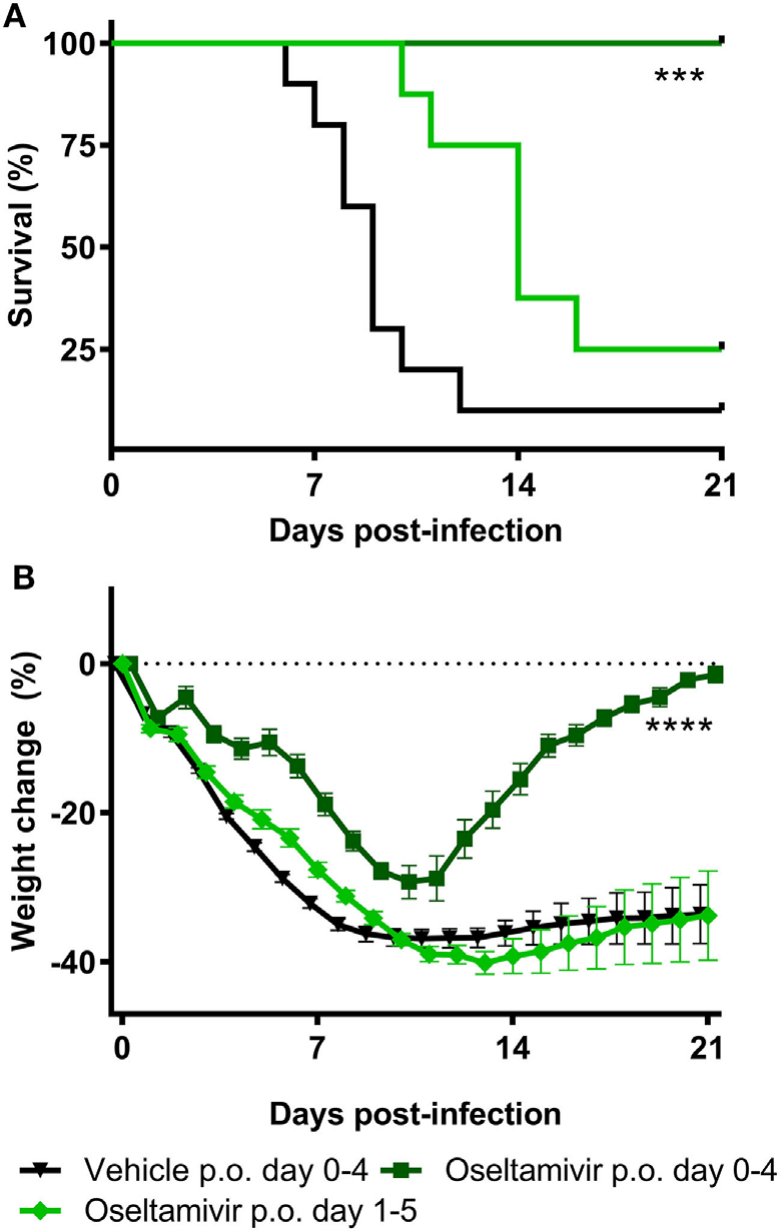
Efficacy of oseltamivir in a lethal BALB/c mouse model. Female BALB/c mice were challenged with a dose of 4MLD_50_ influenza A/Anhui/1/2013 (H7N9) on day 0. Mice received 100 mg/kg twice daily p.o. Oseltamivir treatment from days 0–4 or 1–5 (*n* = 8 per group) or the vehicle control (*n* = 10) from days 0–4. Kaplan–Meyer survival curve **(A)** and mean body weight change **(B)** are depicted. Survival proportion in the oseltamivir treated groups was analyzed using a Fisher’s exact two-sided test with Bonferroni correction for multiple comparisons. The effect of oseltamivir treatment on body weight was analyzed by comparing the area under curve of treatment groups with vehicle and was analyzed using a two-way ANOVA with Bonferroni correction. Error bars depict SEM. ****p < 0.001, ****p < 0.0001.

To determine the sensitivity of the challenge model, we tested the efficacy of the most widely used anti-influenza virus drug, the neuraminidase inhibitor oseltamivir phosphate, as a reference control. Animals were challenged with an intranasal dose of 4MLD_50_ influenza A/Anhui/1/2013 (H7N9). The control group (*n* = 10) was treated with PBS twice daily p.o. for 5 days, starting from day 0. Eight animals were treated twice daily with 100 mg/kg oseltamivir p.o. for 5 days, starting 1 h before challenge at day 0. Another eight mice started on day 1 with 100 mg/kg oseltamivir treatment twice daily for the duration of 5 days. Figure 2A presents the Kaplan–Meier survival curve. In the animals of the vehicle control group, 10% survival was observed after influenza A/Anhui/1/2013 (H7N9) challenge. Treatment with 100 mg/kg oseltamivir twice daily p.o. from day 0 to day 4 resulted in 100% survival, which was significantly improved compared to the vehicle control group (*p*-value < 0.01) (Table S1 in Supplementary Material). Although treatment with 100 mg/kg oseltamivir twice daily p.o. from day 1 to day 5 also resulted in an increased survival proportion of 25%, this was not statistically different from the control group (Table S2 in Supplementary Material). Nevertheless, survival time was significantly increased in both oseltamivir treated groups (treatment day 0–4 *p*-value < 0.01, treatment day 1–5 *p*-value < 0.05) (Table S2 in Supplementary Material). Oseltamivir treatment starting from day 0 showed a significantly reduced body weight loss compared to the control group (*p*-value < 0.001), while initiating oseltamivir treatment 1 day post-challenge, did not reduce the body weight loss compared to the control group (Figure 2B; Table S3 in Supplementary Material).

### BCG Vaccination Prior to H7N9 Infection Challenge

To test the effect of BCG against lethal influenza challenge, a total of 13 mice were BCG immunized and 13 mice were injected with PBS 1 week prior to A/Anhui/1/2013 (H7N9) challenge.

### Cytokine Responses After *Ex Vivo* Splenocyte and Peritoneal Macrophage Restimulation

**Figure 3:**
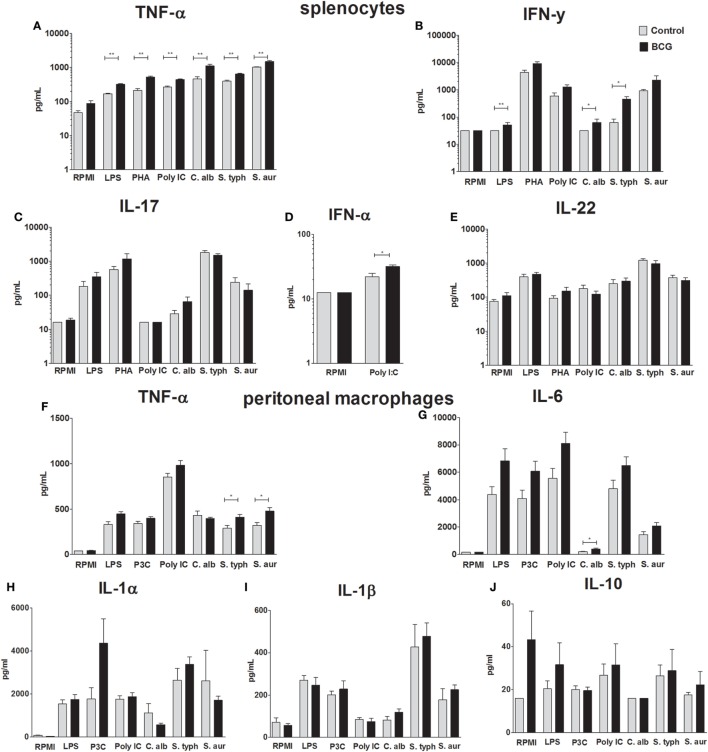
Bacillus Calmette–Guérin (BCG)-induced trained immunity cytokine responses. To demonstrate increased innate immune response after BCG vaccination, spleen cells and peritoneal macrophages were isolated 7 days after administration of either BCG or phosphate buffered saline. Concentrations of tumor necrosis factor-alpha (TNF-α), interferon (IFN)-α, IFN-γ, interleukin (IL)-17, and IL-22 determined by enzyme-linked immunosorbent assay (ELISA) in supernatants of restimulated splenocytes with Roswell Park Memorial Institute (RPMI), lipopolysaccharide (LPS), phytohemagglutin (PHA), poly I:C, *Candida albicans, Salmonella typhi*, and *Staphylococcus aureus* are shown **(A–E)**. Concentrations of IL-1α, IL-1β, IL-6, IL-10, and TNF-α determined by ELISA in supernatants of restimulated peritoneal macrophages with RPMI, LPS, poly I:C, *C. albicans, S. typhi*, and *S. aureus* are shown **(F–J)**. Data are shown as mean ± SEM, *n* = 5 per group, *p < 0.05, **p < 0.01 Mann–Whitney *U* (two-sided).

To assess the systemic trained immunity responses, splenocytes as well as peritoneal macrophages (5 mice per group) were isolated 7 days after BCG immunization. Both cell suspensions were restimulated with several TLR-ligands and pathogens. Cytokines were determined by ELISA in collected supernatants. TNF-α production was significantly increased in the BCG-vaccinated group after restimulation of splenocytes with all stimuli except RPMI medium control (*p*-value < 0.01 for all stimuli) (Figure 3A). Splenocyte-derived IFN-γ responses were significantly upregulated after LPS (*p*-value < 0.01), *C. albicans* (*p*-value < 0.05), and *S. typhi* (*p*-value < 0.05) restimulation (Figure 3B). No significant differences were found on splenocyte-derived IL-17 and IL-22 responses (Figures 3C,E). IFN-α was only determined in supernatants of splenocytes restimulated with RPMI and poly I:C. Restimulation with poly I:C resulted in a small but statistically significantly higher IFN-α response in the BCG-vaccinated group (*p*-value < 0.05) (Figure 3D). Significantly higher TNF-α production in the BCG-immunized mice was found when peritoneal macrophages were restimulated with *S. typhi* (*p*-value < 0.05) and *S. aureus* (*p*-value < 0.05) (Figure 3F). Although a consistent trend in increased IL-6 production was observed when peritoneal macrophages of the BCG-vaccinated group were restimulated with all pathogens and TLR ligands, only stimulation with *C. albicans* resulted in a statistically significant increase in comparison to the control group (*p*-value < 0.05) (Figure 3G). IL-1α, IL-1β, and IL-10 responses (Figures 3H–J) did not differ between groups.

### BCG Vaccination Does Not Protect Mice During H7N9 Influenza Infection

**Figure 4:**
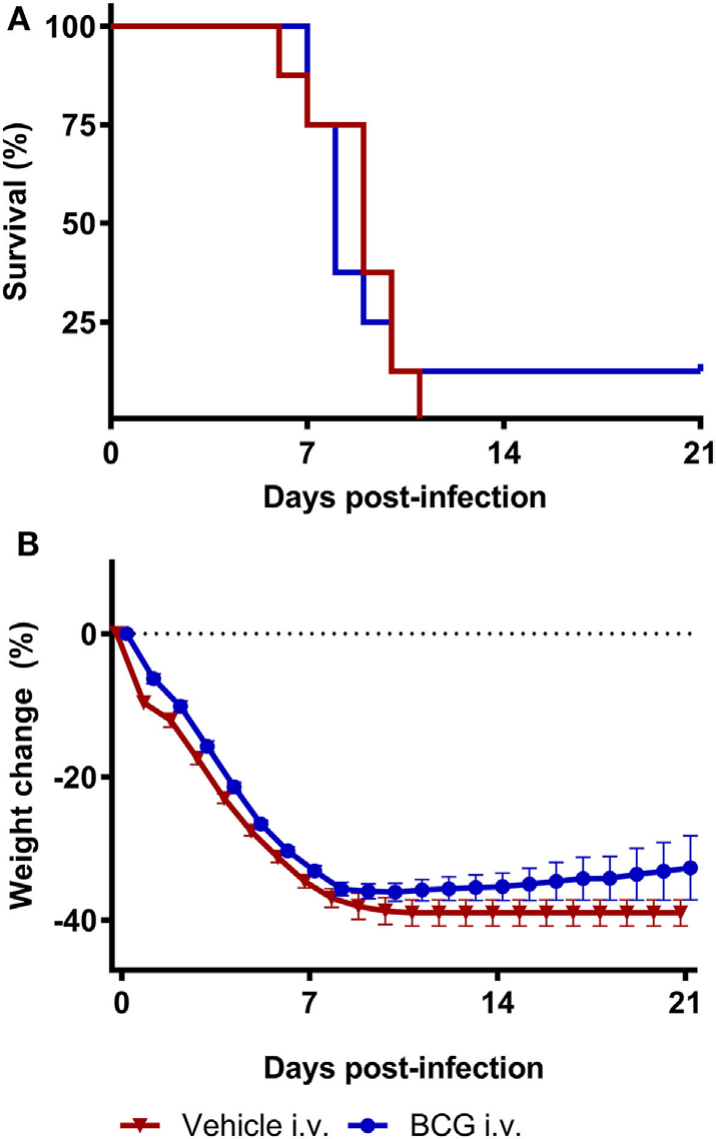
Bacillus Calmette–Guérin (BCG) vaccination does not protect mice during H7N9 influenza infection. Female BALB/c mice were challenged with a dose of 4MLD_50_ influenza A/Anhui/1/2013 (H7N9) on day 0. Mice received 750 µg BCG or the vehicle control (phosphate buffered saline) i.v. on day −7. Kaplan–Meyer survival curve **(A)** and mean body weight change **(B)** are depicted. Survival proportion in the BCG treated group was statistically analyzed using a Fisher’s exact two-sided test. Survival time was statistically analyzed using a Mantel and Cox log-rank test. The effect of BCG treatment on body weight was analyzed by comparing the area under curve of treatment groups with vehicle and was statistically analyzed using a two-way ANOVA with Bonferroni correction. Data are shown as mean ± SEM, *n* = 8 per group.

No survival (0%) was observed in the animals of vehicle control group 11 days after A/Anhui/1/2013 (H7N9) challenge. Despite the heterologous induction of trained immunity responses, as reported above, vaccination with BCG did not result in a statistically significant improvement in survival proportion compared to the vehicle control group (12.5% survival in BCG treated group) (Table S1 in Supplementary Material). Furthermore, BCG vaccination did not result in a statistically significant improvement in survival time compared to the vehicle control group (*p*-value = 0.82) (Figure 4A; Table S2 in Supplementary Material). The percentage body weight change per animal was determined relative to day 0. Similar to the vehicle control group, all animals in the BCG-vaccinated displayed a steep reduction in bodyweight starting from day 1 until day 11. The single surviving animal in the BCG-treated group started to recover after day 11. BCG vaccination did not result in a significant difference in body weight loss compared to the vehicle control group (*p*-value = 0.15) (Figure 4B; Table S3 in Supplementary Material). From day 0 onward, the severity of influenza infection was graded as a clinical score based on the observation of one or multiple clinical signs typical for influenza infection. The mean clinical scores did not differ between the vehicle control group and BCG-immunized group (Figure S1 in Supplementary Material).

### BCG Vaccination Does Not Reduce Histopathological Damage, Inflammation, or Viral Replication

**Figure 5:**
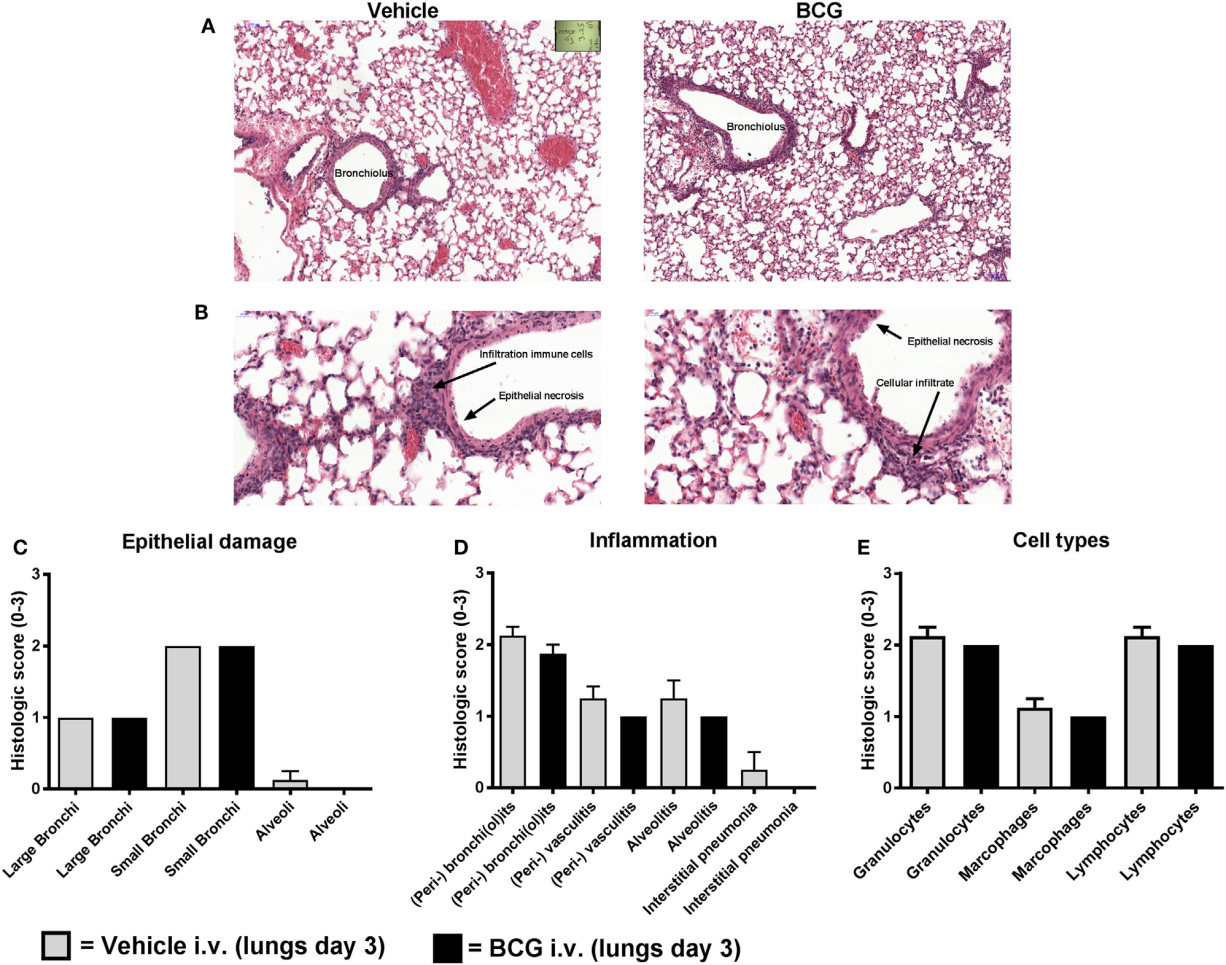
Bacillus Calmette–Guérin (BCG) vaccination does not lead to reduction of histopathological damage and inflammation. Female BALB/c mice were challenged with a dose of 4MLD_50_ influenza A/Anhui/1/2013 (H7N9) on day 0. Mice received 750 µg BCG or vehicle control (phosphate buffered saline) i.v. on day –7 (*n* = 8 per group). Three days after viral challenge, lungs were fixed in formalin. Paraffin-embedded tissue sections were then stained for hematoxylin and eosin. Representative histopathological image of vehicle versus BCG-treated mice are depicted **(A,B)**. Lung sections were scored from absent to marked: score “0” (absent), score “1” (minimal), score “2” (moderate), and score “3” (marked) for epithelial necrosis (damage) **(C)**, inflammation markers **(D)**, and inflammatory cell types **(E)**. Results were statistically analyzed using the Cochran–Mantel–Haenszel test. Data are shown as mean ± SEM.

**Figure 6:**
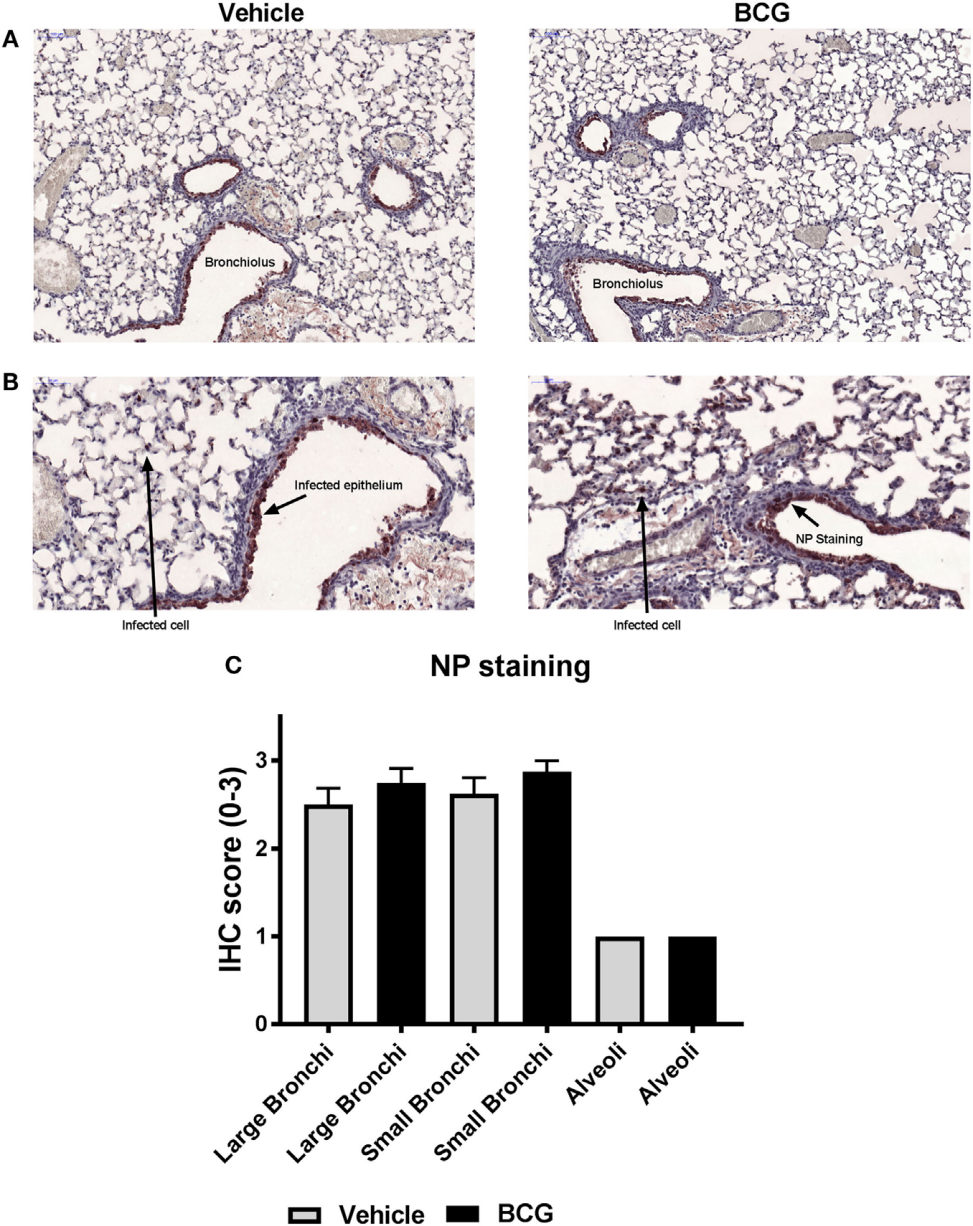
Nucleoprotein (NP) staining of bronchi and alveoli indicates that bacillus Calmette–Guérin (BCG) does not reduce influenza virus replication. Female BALB/c mice were challenged with a dose of 4MLD_50_ influenza A/Anhui/1/2013 (H7N9) on day 0. Mice received 750 µg BCG or the vehicle control (phosphate buffered saline) i.v. on day –7 (*n* = 8 per group). Three days after viral challenge, lungs were fixed in formalin. Paraffin-embedded tissue sections were then stained for influenza NP. Representative images of influenza A NP-stained lungs of mice treated with vehicle versus BCG-treated mice are depicted **(A,B)**. NP-staining was scored from absent to 3: score “0” (absent), score “1” (minimal), score “2” (moderate), and score “3” (marked) **(C)**. Results were statistically analyzed using the Cochran–Mantel–Haenszel test. Data are shown as mean ± SEM.

Three days after challenge infection, histopathological lung examination was performed in a subgroup of both vehicle control and BCG-vaccinated mice. The pathological changes observed in the lungs isolated from these mice corresponded with acute lung injury, characterized by epithelial necrosis and infiltration of macrophages, lymphocytes, and granulocytes (Figures 5A,B). In addition, squamous metaplasia was observed. No differences in pathological changes between the lungs of the BCG vaccinated and vehicle control mice were observed at day 3 after challenge infection (Figures 5C,D). Histological scores for the amount of granulocytes, macrophages, and lymphocytes were similar between the BCG immunized and control group (Figure 5E). Although BCG did not have an effect on pulmonary tissue inflammation or immune cell composition, we wanted to determine the effect of BCG immunization on local influenza infection and replication (by scoring of influenza infected cells). Lung tissue sections slides were stained with influenza NP to determine the quantity of influenza virions (Figures 6A,B). The scores of NP stained small bronchi, large bronchi, or alveoli did not differ between groups (Figure 6C). This indicates that intravenous BCG administration did not reduce influenza A (H7N9) infection or replication in mice.

## Discussion

The avian influenza A (H7N9) virus appears to have become more virulent during recent epidemics in China, underlining the risk of a global human pandemic in the absence of specific immunity. In case of an outbreak in the absence of available specific vaccines, BCG might contribute to control of avian influenza through its non-specific protective effects, which are probably related to its capacity to induce trained immunity in monocytes. In this study, we tested the effect of intravenous BCG against a lethal influenza A/Anhui/1/2013 (H7N9) infection in mice.

We were able to reproduce previous findings regarding induction of systemic trained immunity after BCG administration in mice (16). Intravenous BCG immunization resulted in significantly increased cytokine responses upon *ex vivo* restimulation with unrelated TLR-ligands and pathogens, both in splenocytes (TNF-α, IFN-α, and IFN-γ) and peritoneal macrophage (TNF-α and IL-6). However, increased *ex vivo* cytokine production was not associated with differences in experimental avian influenza A/Anhui/1/2013 (H7N9) infection in terms of survival, clinical scores, or pulmonary inflammation.

A recent study demonstrated that low dose BCG (TICE strain) administration protects against mouse-adapted influenza virus A/Puerto Rico/8/34 (PR8) (H1N1) strain by increased efferocytosis of alveolar phagocytes, but only after i.n. and not after subcutaneously administration of BCG (26). Similar observations were reported previously; i.n. BCG (strain unspecified) immunized mice showed enhanced protection against influenza A (H1N1) PR8 challenge compared to intraperitoneally immunized mice (17). This suggests the route of BCG administration might be important in case of protection against influenza. In the study of Mukherjee et al. (26), mice were inoculated with a lower dose of BCG compared to the administered dose in our study, pointing out that not only the route of administration but maybe also the dose and inherent dose-dependent kinetics might be crucial for heterologous protection. For several TLR-ligands, it has been shown that the dose during initial priming determines if monocytes will either become trained or tolerized during the *in vitro* trained immunity model (27). One could hypothesize that induction of either training or tolerance may be different per cell and tissue type as well. Although *in vivo* dose response studies of BCG priming in the context of trained immunity have not been performed yet, these *in vitro* data indicate the initial dose might be discriminative between being protective or not. That being said, a similar dose, route of administration as well as BCG strain were nevertheless protective against a lethal *C. albicans* challenge infection in severe combined immunodeficient mice, resulting in enhanced survival, decreased kidney yeast burden, and *ex vivo* trained immunity responses (16). This may suggest that the beneficial effects of BCG vaccination have specificity, and thus induce protection against some, but not all, infections.

As reviewed by Kuiken et al. (28), the innate immune system plays an important role in the first line defense against influenza infections. Influenza recognition by TLR and RIG-1 signaling leads to production of pro-inflammatory cytokines and type I IFNs. Especially type I IFNs are known to exert antiviral activity. Nevertheless, an imbalanced cytokine response or so-called cytokine storm could be detrimental for the host. Compared to seasonal strains, severe infections with highly pathogenic H5N1 and 1918 H1N1 are more frequently associated with a dysregulated cytokine response (29, 30). Likewise, severe cases of H7N9 infections are complicated by hypercytokinemia (3, 31, 32). In the study of Mukherjee et al., intranasal BCG inoculation resulted in decreased TNF-α mRNA and increased IL-10 mRNA expression in alveolar macrophages 2 days post PR8 (H1N1) infection (26). We did not study the local pulmonary mucosal cytokine responses after intravenous administration of BCG, but our findings regarding cytokine responses of *ex vivo* restimulated splenocytes and peritoneal macrophages are pointing toward priming in a different direction, with potentiation of cytokine responses. Future studies should focus on the role of alternative cell types like alveolar macrophages.

One unavoidable limitation for the conclusions of this study is that it investigated the effect of BCG vaccination in mice. Anti-mycobacterial as well as anti-influenza responses are different in mice and humans, and it is conceivable to hypothesize that BCG vaccination may exert stronger beneficial effects in humans, including against influenza infection. Recently, we have shown that BCG vaccination in healthy human volunteers protects against experimental yellow fever vaccine viremia, while the protective serological response retains intact (22). The absence of IL-1β upregulation after BCG vaccination by splenocytes and peritoneal macrophages in the current study can be explained by the differences between the human and murine immunological responses. Humans have an increased IL-1β response compared to mice and mice lack important regulators of the IL-1 pathway such as IL-37 (33).

Another limitation of the study is represented by the particularities of the BCG vaccination in mice, compared to humans. Earlier studies done in mice have shown that systemic trained immunity by BCG is induced after intravenous administration, rather than intradermal administration (34). In line with this, an earlier study by Spencer et al. showed protection against influenza A (H1N1) infection after intraperitoneal vaccination with BCG, indicating that systemic administration of BCG was protective against influenza (17). Furthermore, the administered dose of BCG the animals received and the timing of 7 days before the experimental avian influenza A/Anhui/1/2013 (H7N9) infection were equivalent to our previous intravenous BCG vaccination experimental protocols and comparable to the intraperitoneal BCG vaccination of mice (16–18). We hypothesized that in BCG-vaccinated mice, the infiltration of trained immune cells in the lungs during the experimental avian influenza A/Anhui/1/2013 (H7N9) infection would result in increased clearance of the influenza infection. While our data clearly demonstrate that this BCG vaccination model does not protect against H7N9 influenza infection, we cannot exclude that the different route of BCG administration in humans may have an effect.

Partial protection of BCG vaccination has been observed in a human-controlled malaria infection model as well, in which decreased parasitemia correlated with accelerated immune activation and trained immunity *in vivo* upon blood stage parasitemia (Walk, de Bree et al., submitted). A previous randomized controlled trial has shown BCG vaccination prior to trivalent influenza vaccination resulted in enhanced immunogenicity of the influenza vaccine, resulting in increased antibody response and seroconversion against the 2009 pandemic influenza A (H1N1) strain (21). Future studies should explore if BCG could function as an adjuvant to avian influenza vaccines as well.

In conclusion, intravenous administration of BCG enabled the induction of trained immunity, but this was not protective in a lethal influenza A/Anhui/1/2013 (H7N9) challenge infection in BALB/c mice. Future studies are needed to evaluate the anti-influenza effects of BCG vaccination in humans.

## Ethics Statement

Animals experiments were performed in accordance with the guidelines of the European Communities (Directive 2010/63/EU) and Dutch legislation (The experiments on Animals Act, 1997). The animal experimental protocols were approved by an independent Animal Ethics Committee (TNO, Zeist, the Netherlands) under project license 3387 and performed in the AAALAC accredited animal facility of Triskelion. All animals were housed in a temperature and light-cycle controlled facility with unlimited access to food and water. All procedures involving live H7N9 viruses, including the animal experiments, were carried out in a BSL-3 containment facility at Triskelion. The animals were monitored for clinical signs of influenza disease twice daily with intervals of at least 5 h. All observations, including behavioral aspects were recorded. If lethargy was observed longer than 48 h, the animal was euthanized (humane endpoint).

## Author Contributions

Participated in research design: RM, JD, MN, and LJ. Conducted experiments: RM and JK. Performed data analysis: RM, SH, MW, and LdB. Wrote the manuscript: LdB and RM. Critically read the manuscript: PA, RC, CSB, DD, JD, MW, MN, LJ, SH, and JK.

## Conflict of Interest Statement

JD is currently employed by Aduro Biotech, but was employed by Triskelion when the studies were performed. All other authors declare no competing interests. The reviewer CL and handling Editor declared their shared affiliation.

## Abbreviations

BCG, bacillus Calmette–Guérin; DMEM, Dulbecco’s Modified Eagle Medium; ELISA, enzyme-linked immunosorbent assay; IL-6, interleukin-6; IL-10, interleukin-10; IFN, interferon; LPS, lipopolysaccharide; MLD, mouse lethal dose; NP, nucleoprotein; PBMC, peripheral blood mononuclear cell; PHA, phytohemagglutin; RPMI, Roswell Park Memorial Institute; TCID, tissue culture infective dose; Th1, T-helper cell 1; Th17, T-helper cell 17; TLR, toll-like receptor; TNF-α, tumor necrosis factor-alpha.
