# Corrigendum: Bacillus Calmette–Guérin-Induced Trained Immunity Is Not Protective for Experimental Influenza A/Anhui/1/2013 (H7N9) Infection in Mice

**DOI:** 10.3389/fimmu.2018.02471

**Published:** 2018-10-25

**Authors:** L. Charlotte J. de Bree, Renoud J. Marijnissen, Junda M. Kel, Sietske K. Rosendahl Huber, Peter Aaby, Christine Stabell Benn, Marcel V. W. Wijnands, Dimitri A. Diavatopoulos, Reinout van Crevel, Leo A. B. Joosten, Mihai G. Netea, John Dulos

**Affiliations:** ^1^Department of Internal Medicine, Radboud University Medical Center, Nijmegen, Netherlands; ^2^Radboud Centre for Infectious Diseases (RCI), Radboud University Medical Center, Nijmegen, Netherlands; ^3^Research Center for Vitamins and Vaccines, Bandim Health Project, Statens Serum Institut, Copenhagen, Denmark; ^4^Odense Patient Data Explorative Network, University of Southern Denmark, Odense University Hospital, Odense, Denmark; ^5^Department of Immunology, Triskelion B.V., Zeist, Netherlands; ^6^Laboratory of Pediatric Infectious Diseases, Radboud Institute for Molecular Life Sciences, Radboud University Medical Center, Nijmegen, Netherlands; ^7^Department for Genomics and Immunoregulation, Life and Medical Sciences Institute (LIMES), University of Bonn, Bonn, Germany; ^8^Aduro Biotech Europe, Oss, Netherlands

**Keywords:** avian influenza A/Anhui/1/2013 (H7N9), bacillus Calmette–Guérin, trained immunity, innate immune memory, oseltamivir

An author name was incorrectly spelled as Charlotte L. C. J. de Bree. The correct spelling is L. Charlotte J. de Bree. The authors apologize for this error and state that this does not change the scientific conclusions of the article in any way. The original article has been updated.

## Conflict of interest statement

JD is currently employed by Aduro Biotech, but was employed by Triskelion when the studies were performed. The remaining authors declare that the research was conducted in the absence of any commercial or financial relationships that could be construed as a potential conflict of interest.

