# Supplementary figures and images for: Bacillus Calmette–Guérin-Induced Trained Immunity Is Not Protective for Experimental Influenza A/Anhui/1/2013 (H7N9) Infection in Mice
Source: Front Immunol. 2018 Apr 30;9:869. doi: 10.3389/fimmu.2018.00869 (PMC5936970; doi:10.3389/fimmu.2018.00869)

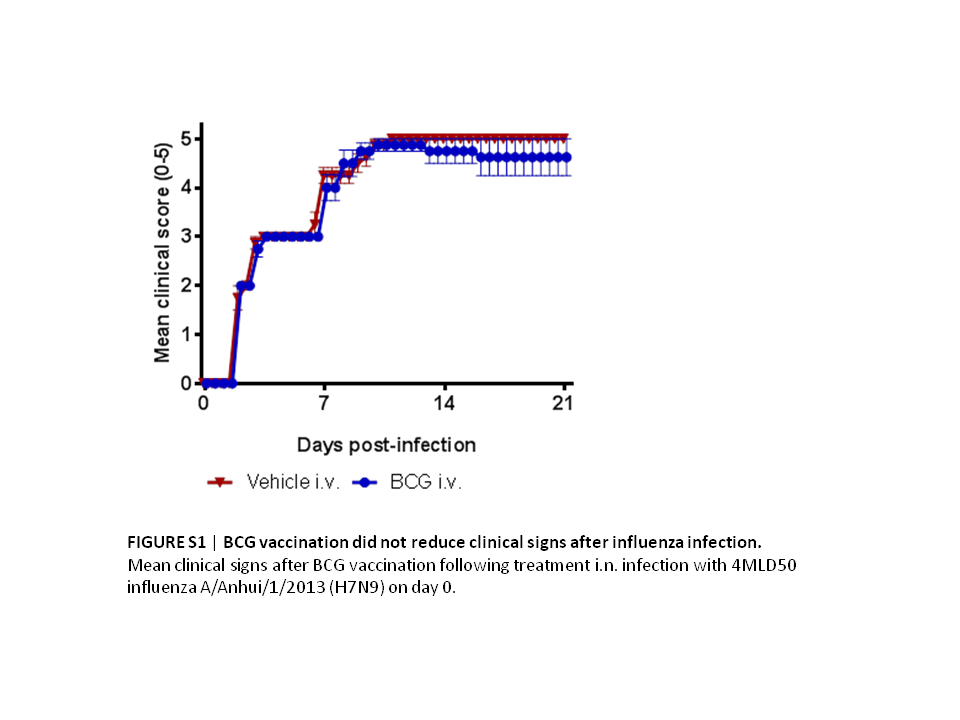

Supplement: Supplementary file 1 [file Image_1.tif]
