# Supplementary material for: Bacillus Calmette–Guérin-Induced Trained Immunity Is Not Protective for Experimental Influenza A/Anhui/1/2013 (H7N9) Infection in Mice
Source: Front Immunol. 2018 Apr 30;9:869. doi: 10.3389/fimmu.2018.00869 (PMC5936970; doi:10.3389/fimmu.2018.00869)
Supplement: Supplementary file 2 [file Table_1.docx]

**TABLE S1** | **Survival proportion.**

The treatment effect in the influenza challenge model was assessed by comparing the survival proportions at day 21 of each treatment group to the vehicle control group, using a Fisher’s exact two-sided test, with Bonferroni adjustment for multiple comparisons. A *p*-value <0.05 was considered as statistically significant.

| **Treatment** | **Survival proportion**  **%** | **Adjusted *p*-value for comparison with control group** |
| --- | --- | --- |
| Vehicle p.o. day 0-4 | 10 | - |
| Oseltamivir p.o. day 0-4 | 100 | 0.0004 |
| Oseltamivir p.o. day 1-5 | 25 | 1 |
|  |  |  |
| Vehicle i.v. | 0 | - |
| BCG i.v. | 12.5 | 1 |
