# Supplementary material for: Bacillus Calmette–Guérin-Induced Trained Immunity Is Not Protective for Experimental Influenza A/Anhui/1/2013 (H7N9) Infection in Mice
Source: Front Immunol. 2018 Apr 30;9:869. doi: 10.3389/fimmu.2018.00869 (PMC5936970; doi:10.3389/fimmu.2018.00869)
Supplement: Supplementary file 3 [file Table_2.docx]

**TABLE S2** | **Survival time.** The treatment effect in the influenza challenge model was assessed by comparing the survival times to the vehicle control group using a Mantel & Cox Log-Rank test with Bonferroni adjustment for multiple comparisons. A *p*-value <0.05 was considered as statistically significant. The survival time of an animal was defined as the last study day on which it was alive.

| **Treatment** | **Mean survival time (days)** | **P-value** |
| --- | --- | --- |
| Vehicle p.o. day 0-4 | 8.3 | - |
| Oseltamivir p.o. day 0-4 | 21.0 | 0.0004 |
| Oseltamivir p.o. day 1-5 | 15.0 | 0.03 |
|  |  |  |
| Vehicle i.v. | 8.9 | - |
| BCG i.v. | 9.8 | 0.82 |
