# Supplementary material for: Bacillus Calmette–Guérin-Induced Trained Immunity Is Not Protective for Experimental Influenza A/Anhui/1/2013 (H7N9) Infection in Mice
Source: Front Immunol. 2018 Apr 30;9:869. doi: 10.3389/fimmu.2018.00869 (PMC5936970; doi:10.3389/fimmu.2018.00869)
Supplement: Supplementary file 4 [file Table_3.docx]

**TABLE S3** | **Area under curve body weight.**

The treatment effect in the influenza challenge model was assessed by comparing the AUCs of each treatment group to the negative vehicle control group using a one-way analysis of variance, tested two-sided, with Bonferroni adjustment for multiple comparisons. Last observed body weights were carried forward. A *p*-value <0.05 was considered as statistically significant.

| **Treatment** | **Mean AUC** | **SD** | **P-value** |
| --- | --- | --- | --- |
| Vehicle p.o. day 0-4 | -115.64 | 14.67 | - |
| Oseltamivir p.o. day 0-4 | -52.90 | 10.74 | <0.0001 |
| Oseltamivir p.o. day 1-5 | -116.35 | 21.14 | 1 |
|  |  |  |  |
| Vehicle i.v. | -128.59 | 16.63 | - |
| BCG i.v. | -115.11 | 18.62 | 0.15 |
